# Colorectal cancer-associated *Streptococcus infantarius* subsp. *infantarius* differ from a major dairy lineage providing evidence for pathogenic, pathobiont and food-grade lineages

**DOI:** 10.1038/s41598-018-27383-4

**Published:** 2018-06-15

**Authors:** Dasel Wambua Mulwa Kaindi, Wambui Kogi-Makau, Godfrey Nsereko Lule, Bernd Kreikemeyer, Pierre Renault, Bassirou Bonfoh, Nize Otaru, Thomas Schmid, Leo Meile, Jan Hattendorf, Christoph Jans

**Affiliations:** 1 Department of Food Science, Nutrition and Technology, University of Nairobi, Nairobi, Kenya; 2 School of Medicine, University of Nairobi, Nairobi, Kenya; 3 Institute of Medical Microbiology, Virology, and Hygiene, Rostock University Medical Centre Rostock, Rostock, Germany; 4 Institut National de la Recherche Agronomique, UMR 1319 MICALIS, Jouy-en-Josas, France; 5 Centre Suisse de Recherches Scientifiques en Côte d’Ivoire, Adiopodoume, Ivory Coast; 6 Department of Epidemiology and Public Health, Swiss Tropical and Public Health Institute, Basel, Switzerland; 7 University of Basel, Basel, Switzerland; 8 Laboratory of Food Biotechnology, ETH Zurich, Zurich, Switzerland

## Abstract

*Streptococcus infantarius* subsp. *infantarius* (*Sii*), a member of the *Streptococcus bovis/Streptococcus equinus* complex (SBSEC), predominates as dairy-adapted and non-adapted variants in fermented dairy products (FDP) in East and West Africa. Epidemiologic data suggest an association with colorectal cancer for most SBSEC members, including *Sii* from Kenyan patients. Phylogenetic relationships of East African human (EAH) isolates to those of dairy and pathogenic origin were analysed to better estimate potential health implications via FDP consumption. The MLST-derived population structure was also evaluated to provide host, disease, geography and dairy adaptation associations for 157 SBSEC isolates, including 83 novel *Sii*/SBSEC isolates of which 40 originated from Kenyan colonoscopy patients. Clonal complex (CC) 90 was delineated as potential pathogenic CC for *Sii*. Single EAH, West African dairy (WAD), food and animal *Sii* isolates clustered within CC-90, suggesting a potential link to pathogenic traits for CC-90. The majority of EAH and WAD *Sii* were clustered in a shared clade distinct from CC-90 and East African dairy (EAD) isolates. This indicates shared ancestry for the EAH and WAD clade and limitations to translate disease associations of EAH and CC-90 to EAD *Sii*, which could support the separation of pathogenic, pathobiont/commensal and food lineages.

## Introduction

*Streptococcus infantarius* subsp. *infantarius* (*Sii*) belongs to the *Streptococcus bovis*/*Streptococcus equinus* complex (SBSEC). SBSEC is a diverse group of bacteria including commensal inhabitants of the human and animal gastrointestinal tract, opportunistic pathogens and variants in food^1,2^. The SBSEC is composed of seven subspecies: *Streptococcus gallolyticus* subsp. *gallolyticus* (*Sgg*), *Streptococcus gallolyticus* subsp. *macedonicus (Sgm), Streptococcus gallolyticus* subsp. *pasteurianus* (*Sgp*)*, Streptococcus equinus, Streptococcus infantarius* subsp*. infantarius (Sii), Streptococcus lutetiensis* and *Streptococcus alactolyticus*. SBSEC provides enhanced discrimination power in contrast to the former *Streptococcus bovis* designation that is still used for its wide recognition^2,3^.

SBSEC group members are associated with bacteraemia, infective endocarditis, urinary tract infections, meningitis, sepsis, gastroenteritis, endophthalmitis and carcinoma of the colon^4–6^. *Sgg* feature a strong association with colorectal cancer, particularly in patients affected by *Sgg*-related bacteraemia or infective endocarditis^7^. To a lesser extent, this association is also observed for *S. lutetiensis*, *Sgp* and possibly even *Sii* and *Sgm*. However, the causality of SBSEC members in colorectal cancer is still questioned and rather explained by a bacterial driver-passenger model^7–10^. In contrast to *Sgg*, *Sgp* and *S. lutetiensis*, *Sii* is a rare isolate in clinical specimens^11,12^, partially because *Sii* was only recently separated from *S. lutetiensis* (former *S. infantarius* subsp. *coli*) within the *S. infantarius* branch^2,3^. This separation provides clarity regarding the involvement of subspecies of *S. infantarius* in disease that was rarely available in studies conducted prior to this new taxonomy. *Sii* is implicated in human infections including infective endocarditis^13^, biliary tract infection, cirrhotic bacteraemia and non-colorectal cancer^8,10,14–18^.

The habitat of *Sii* as well as most other SBSEC members is mainly in the gastrointestinal tract of humans as well as animals such as ruminants, sea otters and birds^2,3^, where they are considered to be commensals but also fit as putative representatives of pathobionts^19^. Furthermore, *Sii* and *Sgm* are regularly found as predominant organisms in fermented dairy products (FDP)^2^. Particularly, *Sii* is highly prevalent as a predominant bacterium in traditional FDP in sub-Saharan Africa^20–23^. Thus, live *Sii* are ingested by millions of FDP consumers in sub-Saharan Africa at levels over 10^8^ CFU mL^−1^. However, East African and many West African dairy *Sii* are clearly different from the human *Sii* analysed to date^24–26^. African dairy *Sii* feature dairy adaptations such as a modified lactose metabolism via a *lacS* and *lacZ* encoded lactose uptake instead of the SBSEC-typical lactose phosphotransferase system^24,25^. Phylogenetic analysis further supports the differentiation of specific African dairy lineages by multi locus sequence typing (MLST)^24^. Several of these *Sii* lineages seem to be clearly separating from human commensal and human pathogenic lineages while other dairy isolates share a closer relationship with potentially harmful strains^24^.

Knowledge of phylogeny of human commensal and pathogenic lineages is currently limited to strains isolated from humans in Europe and Asia; analogous knowledge of African *Sii* is limited to dairy strains only^24^. Representative human *Sii* isolates from African countries such as Kenya, Somalia or Côte d’Ivoire with a documented presence of dairy *Sii* were missing and therefore did not allow for a more comprehensive phylogenetic evaluation of lineages, strains and their relationships. In a recent hospital-based study on patients undergoing colonoscopy at Kenyatta National Hospital in Nairobi, Kenya, strains to investigate this missing link were obtained^27^. Among these colonoscopy patients, *Sii* and other SBSEC members were isolated from faecal samples and rectal swabs, and in conjunction, the socioeconomic aspects and dietary habits of the patients were assessed. The result was a set of isolates that included comprehensive descriptions of the patients^27^. In that study, SBSEC and *Sii* carriage rate among 273 participants was about 20%^27^. Furthermore, *Sii* isolates among these study participants indicated associations with colorectal cancer and haemorrhoids^27^. However, the population structure and relationships to human and dairy strains is unknown, leaving an important knowledge gap in the epidemiology of *Sii*-associated diseases and the role of dairy *Sii* in Africa, particularly in the example of Kenya. Given the large number of daily consumers of FDP and thus *Sii* consumers in Kenya, closing this knowledge gap of dairy vs human *Sii* in Kenya also has a significant public health impact.

Therefore, the objective of this study was to evaluate the phylogenetic relationships among the first African human *Sii* and other SBSEC members in comparison with dairy, commensal and pathogenic Eurasian strains through the SBSEC-specific MLST approach. Thereby, we investigated the evolution of *Sii* and SBSEC with a global and an African perspective. Particularly for Africa, a thorough safety assessment of dairy *Sii* and their relationship to potentially pathogenic and human commensal lineages is of high priority, given the role of *Sii* in the daily diet of millions of pastoralists in sub-Saharan Africa^28^. This study represents the first phylogenetic analysis of African human *Sii* and SBSEC isolates.

## Results

### MLST analysis of novel SBSEC isolates and update on key characteristics of the SBSEC MLST scheme

**Figure 1:**
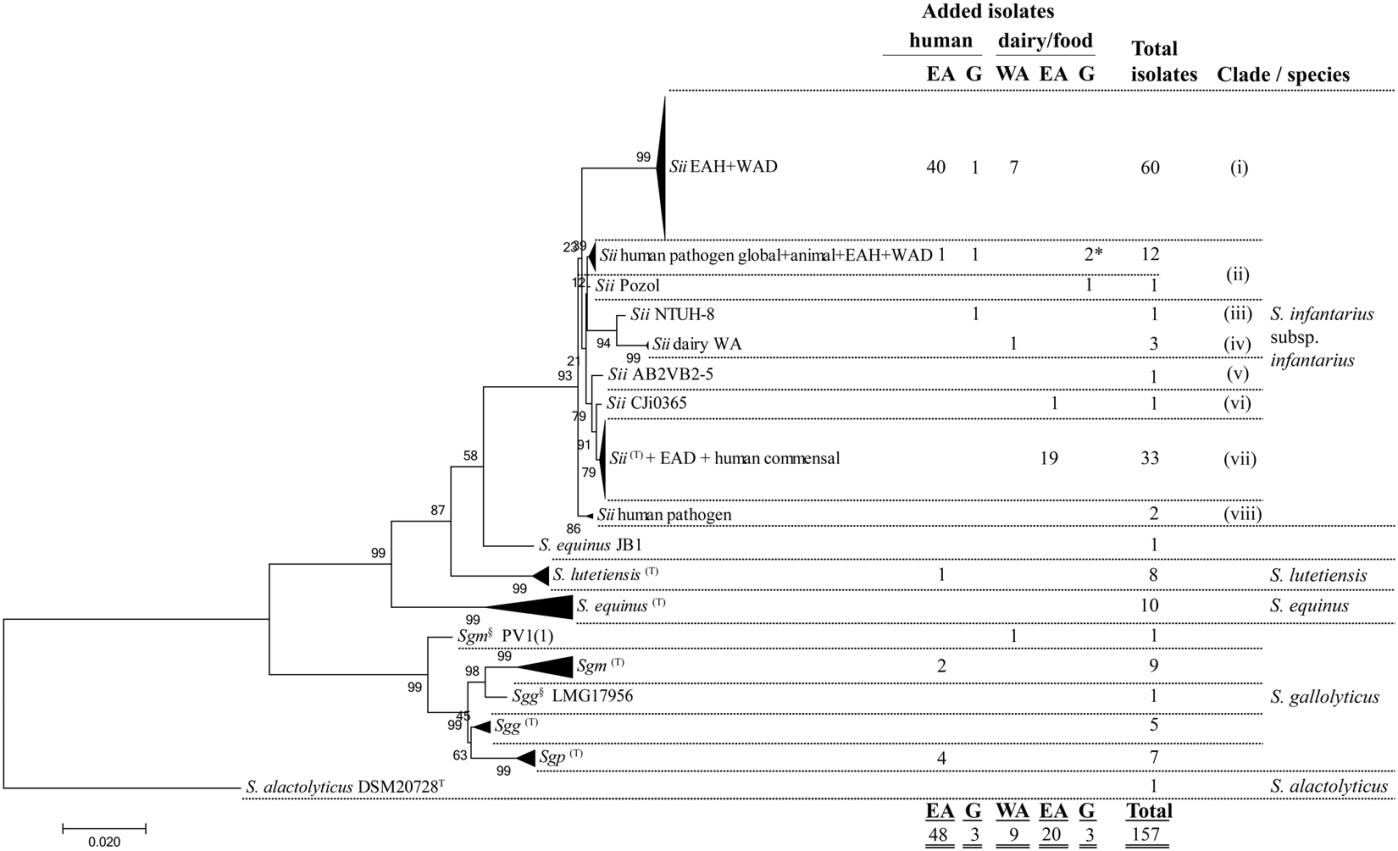
Phylogenetic MLST tree of 157 SBSEC isolates including 83 new isolates. Maximum likelihood phylogenetic tree calculated from the concatenated sequences of the 10 MLST loci of 157 SBSEC isolates including a total of 83 new isolates of East African (EA) human, EA dairy, West African (WA) dairy and global (G) isolates of human, dairy, food or animal origin. The tree is rooted to *S. alactolyticus* DSM20728^T^. Validation was performed using 200 bootstrap replications for which the percentage of clustered trees is given next to the branches. The horizontal bar at the bottom indicates the evolutionary distance in the same units as used for branch length. * includes one isolate of animal origin; ^§^marks strains with species designations according to *groEL* sequence but tree position not within the expected subspecies clades.

This study marks the first comparison of human SBSEC and *Sii* isolates of African origin to those of human commensal, human pathogen, animal commensal and dairy origin of a global collection. A total of 83 SBSEC and *Sii* isolates were integrated into the SBSEC multi locus sequence typing (MLST) scheme to yield 157 isolates for the subsequent assessment of evolutionary and phylogenetic relationships (Fig. 1). All isolates originating from human, animal and food collections were previously identified as SBSEC members using SBSEC-specific PCR assays targeting the 16S rRNA gene and to species level using partial *groEL* sequencing^6,20,21,24,27,29–33^.

The SBSEC MLST scheme currently contains 120 STs out of these 157 isolates (Table 1).

**Table 1:** Key characteristics of the SBSEC-MLST scheme for 157 SBSEC isolates calculated for the overall complex and subspecies with newly incorporated alleles.

	SBSEC	n = 157		*Sii*	n = 114		*S. lutetiensis*	n = 9		*Sgm*	n = 10		*Sgp*	n = 7	
	I_A_ = 2.784		d_N_/d_S_	I_A_ = 1.864		d_N_/d_S_	I_A_ = 1.959		d_N_/d_S_	I_A_ = 2.195		d_N_/d_S_	I_A_ = 0.534		d_N_/d_S_
	I_A_^S^ = 0.309			I_A_^S^ = 0.207			I_A_^S^ = 0.218			I_A_^S^ = 0.244			I_A_^S^ = 0.059		
	ST and alleles			ST and alleles			ST and alleles			ST and alleles			ST and alleles		
	New	Total		New	Total		New	Total		New	Total		New	Total	
ST	54	120		47	78		1	9		3	10		3	6	
*ddl*	10	38	0.080	8	13	0.100	0	6	0.149	2	4	0.121	1	3	0.072
*gki*	7	34	0.057	4	9	0.125	0	5	0.089	3	6	1.368	1	2	0.328
*glnA*	6	27	0.038	4	11	0.129	0	6	0.080	1	2	0.141	0	3	0
*mutS*	7	37	0.028	4	9	0.213	0	5	0.600	2	6	0.065	1	3	0.146
*mutS2*	11	47	0.030	8	18	0.179	0	5	0	3	5	0	1	3	0.036
*pheS*	8	36	0.023	5	15	0.011	0	4	0.046	1	3	0.017	0	3	0
*proS*	12	40	0.063	9	16	0.322	1	6	1.089	2	3	0.398	2	2	0
*pyrE*	30	66	0.022	22	38	0.043	1	6	0	1	4	0.058	1	2	0
*thrS*	4	35	0.082	3	14	0.107	0	5	0.074	2	4	0.099	1	2	0.140
*tpi*	7	24	0.148	4	8	0.761	0	5	0	2	4	0.575	0	2	0

New alleles include all new alleles incorporated into the SBSEC MLST scheme. For individual species, the number corresponds to new alleles assigned to a species, which includes also existing SBSEC alleles that were previously not assigned to a specific. I_A_: index of association; I_A_^S^: standardized index of association; d_N_/d_S_: ratio of non-synonymous sites over synonymous sites.

MLST analysis confirmed the subspecies of all 83 isolates, including 75 *Sii*, three *Sgm*, four *Sgp*, and one *S. lutetiensis* previously determined by *groEL* sequencing (Table 1). The new isolates were responsible for a total of 54 new STs of which 47 were due to *Sii* isolates in addition to one *S. lutetiensis*, three *Sgm* and three *Sgp* isolates as a result of four to 30 new alleles for the 10 different loci (Table 1). I_A_ and I_A_^S^ were calculated to be 0.534–2.784 and 0.059–0.309, respectively, indicating significant linkage disequilibrium for most species except *Sgp* (Table 1). The values of d_N_/d_S_ < 1 indicate no selective pressure for most alleles except for *gki* in *Sgm* (Table 1). However, while overall SBSEC (n = 157) and *Sii* (n = 114) provided sufficient data for calculations, the number of alleles for *S. lutetiensis*, *Sgm* and *Sgp* is still comparably low for reliable calculations.

### Phylogenetic analysis of *Sii* by MLST in relation to host, source, geography and genetic markers

A total of 114 *Sii* isolates were included in this analysis, of which 41 originated from human faecal samples and rectal swabs obtained in Kenya, referred hereafter as East African human (EAH) isolates (colonoscopy patient *Sii* isolates n = 34, infant faecal sample *Sii* isolates n = 7). Four out of the 33 Kenyatta National Hospital (KNH) patients delivered *Sii* isolate pairs. The isolate pairs of three patients were determined as potential isolate duplicates per patient (D261/D933, D1201/D1202, D1396/D1437), whereas one patient delivered two unique isolates D1095 and D1098 (Supplementary Fig. S1).

The *Sii* were separated into eight distinct clades with the major clusters comprised of (i) isolates of West African Dairy (WAD) and EAH, (ii) isolates of human blood (pathogenic), animals, EAH, WAD and pozol; and (vii) isolates of East Africa dairy (EAD) origin as well as the *Sii* type strain and presumptive commensal *Sii* (Fig. 1 and Supplementary Fig. S1). *Sii* strains added in this study were clustered in all *Sii* clades except clade (v).

Geography and sample origin were observed as important aspects for clade structure in relation to dairy isolates. WAD and EAD *Sii* isolates analysed in this study clustered predominantly in the clades (i) and (vii), respectively. In contrast, EAH isolates clustered in clade (i) with the exception of isolate D1266, which clustered in clade (ii). EAH *Sii* isolates obtained from colonoscopy patients at KNH, Nairobi, Kenya (33 isolates) and from infant faecal samples in Msambweni, Kenya (7 isolates), clustered in clade (i) shared with WAD/Asian *Sii* isolates (19 isolates), whereas one EAH *Sii* isolate (D1266) clustered in clade (ii) of human pathogens (Fig. 1 and Supplementary Fig. S1). Subdivisions of individual clades were not further followed due to limited bootstrap reliability.

Analysis of alleles and loci revealed that clustering of WAD/Asian *Sii* (n = 19) with EAH *Sii* (n = 40) in clade (i) was due to shared or highly similar alleles for *glnA*, *mutS2*, *proS*, *thrS* and *tpi*, despite different geographical origins and biological sources. The consensus allele array (*ddl, gki, glnA, mutS, mutS2, pheS, proS, pyrE, thrS, tpi*) for WAD and EAH was (2,2,2,6,2,2,2,3,2,2) and (3,3,2,1,2,3,2,41,2,2), respectively. This consensus is clearly different from that of EAD isolates and human commensal isolates of clade (iii) featuring the consensus allele array (1,1,1,4,6,1,1,1,4,1). DNA sequence analysis of all shared alleles (*glnA*, *mutS2*, *proS*, *thrS* and *tpi*) among WAD and EAH isolates in clade (i) displayed highest sequence identity to other *Sii* isolates for *glnA*, *mutS2*, *proS* and *tpi*. In contrast, *thrS* allele sequences of all *Sii* isolates of clade (i) featured highest sequence identity to *S. thermophilus*. This highest sequence identity to *S. thermophilus* was observed for all *thrS* alleles (2, 8, 18, 32 and 34) comprised in isolates of clade (i), and confirms previous indications of potential horizontal gene transfer (HGT) in this clade for *thrS*^24^. In addition, *thrS* displayed also characteristics of HGT among other SBSEC members. *thrS* alleles 23 and 31, as well as 33, shared highest sequence identity with *S. salivarius* and *S. thermophilus*, respectively. Alleles 23 and 33 were observed in Kenyan *Sgm* isolates of human origin and *Sgm* DSM15879^T^ from Greek cheese. Allele 31 was present in *S. alactolyticus* DSM 20728 ^T^.

**Figure 2:**
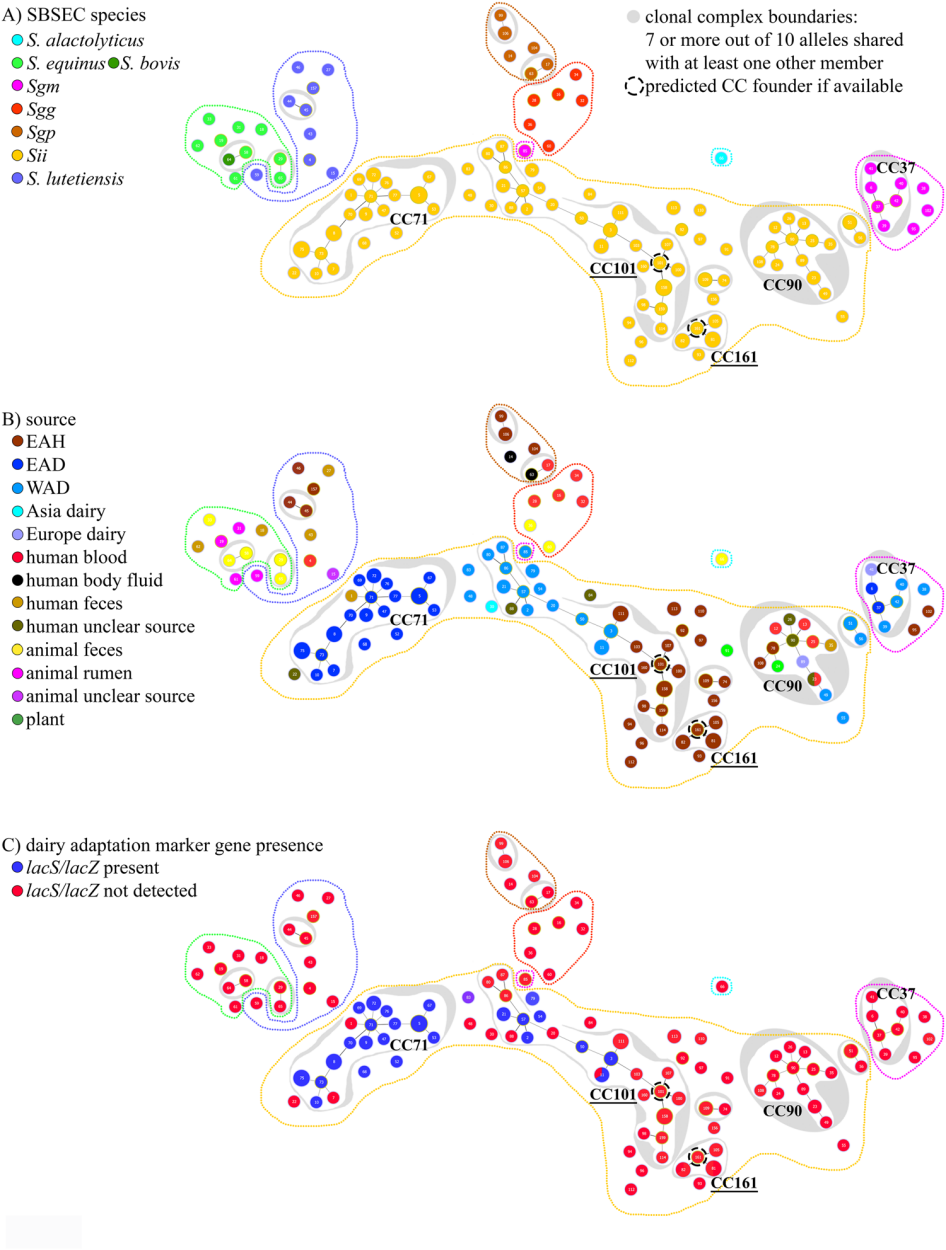
Schematic relationships of SBSEC isolates by MLST sequence types coloured according to isolate metadata. Philoviz/eBURST clustering of SBSEC MLST sequence types (STs) of human, animal and dairy sources calculated from ST profiles. ST bullet points were coloured according to schemes for (**A**) species and subspecies tree including dotted lines in the same colour for graphs A-C for species and subspecies; (**B**) origin and source of isolates and STs; and (**C**) presence of dairy adaptation marker genes *lacS* and *lacZ*. Clustering was initially calculated using eBURST algorithm set to fullMST specifications in Phyloviz 2.0 and subsequently reduced to clonal complex (CC) definition of 7 or more alleles shared with at least one other member of the CC to yield this figure. For CC with >2 STs and predicted founder, the CC is labelled by underlined font. ST bullet point positions and cluster layout correspond to nearest phylogenetic relationships with the exception of *S. alactolyticus* DSM 20728^T^ (ST66), which was placed outside of other species for improved visibility. ST bullet point size corresponds to the number of isolates for each specific ST with the smallest bullet points representing single isolates.

Clonal Complex (CC) calculations, with a CC definition based on STs sharing seven or more out of 10 alleles^24^, revealed six CCs for *Sii*, two for *S. equinus/S. bovis*, two for *Sgp* and one each for *S. lutetiensis* and *Sgm* (Fig. 2). Among these, five CCs were comprised of more than two STs and were labelled CC-101, CC-71, CC-90 and CC-161 for *Sii* and CC-37 for *Sgm*. Out of the *Sii* CCs, CC-101 was comprised of 32 isolates yielding 21 different sequence types (STs) (Fig. 2 and Supplementary Fig. S1). The predicted founder of CC-101 was ST 101. The other *Sii* CC-71, CC-90 and CC-161 were comprised of 17, 12 and 4 STs representing 31, 13 and 10 isolates that were centred around ST71, ST90 and ST161, respectively. However, a founding ST prediction was only possible for ST161 in CC-161, while for the CCs comprising ST71 or ST90, no founding ST could be calculated.

Origin and source were strongly associated with *Sii* CCs (Fig. 2). CC-90 encompassed all *Sii* human blood isolates of potential pathogenic backgrounds from worldwide sources, human clinical isolates of unknown body sites as well as several EAH, WAD, European dairy and plant isolates, raising safety concerns for members of CC-90. CC-71 was predominantly comprised of EAD *Sii* isolates. EAD *Sii* isolates and STs were all comprised within CC-71 with the exception of two singletons. CC-101 was a mix of WAD and EAH *Sii* isolates, including a Spanish hospital isolate (ST88). In contrast to the focused clustering of EAD *Sii* isolates in CC-71, WAD and EAH *Sii* isolates displayed wider distribution among three different CC-s and numerous singletons. This higher diversity of WAD vs. EAH and EAD *Sii* isolates was supported by SID analysis of the *Sii* STs for EAD, EAH and WAD of 0.940, 0.970 and 0.975, respectively, and also for the individual ten alleles (Supplementary Table S1). The higher SID of EAH and WAD *Sii* was mainly due to isolates being spread over different clades. Focusing only on clade (i), SID indicated a lower diversity of 0.968 and 0.961 for EAH and WAD *Sii*, respectively (Supplementary Table S1). Similarly, sequence identity analysis of the concatenated sequences of the 10 alleles of all EAH *Sii* isolates featured an average identity of 99.6% vs. 99.8 for EAD and 98.7 for WAD *Sii* (Supplementary Table S2). Within clade (i), EAH and WAD *Sii* featured 99.7% identity, showing that within a clade, sequence conservation was rather high (Supplementary Table S2).

The presence of the dairy adaptation marker genes *lacS/lacZ* was strongly associated with *Sii* CCs and MLST clades (Supplementary Fig. S1 and Fig. 2). Marker genes were only detected in EAD and WAD *Sii* isolates. None were detected in EAH *Sii* isolates (Supplementary Fig. S1). EAD *Sii* isolates shared 96.9% prevalence of *lacS/lacZ* genes among 32 isolates. In contrast, WAD *Sii* isolates (n = 24) only presented 54.2% *lacS/lacZ* prevalence that was focused on isolates clustered in four small clades and two single branches. In relation to CCs, CC-71 was mainly comprised of EAD *Sii* isolates featuring *lacS/lacZ* adaptation in 15 out of 17 STs, with *Sii* type strain (ST1, human faecal isolate) and *Sii* 150A (ST7, EAD isolate) being the exceptions. CC-101 including WAD *Sii* isolates only presented *lacS/lacZ* in six out of 11 WAD STs, but not in any of the EAH STs. Similarly, no *lacS/lacZ* adaptation was detected in human blood isolates or any STs of CC-90 and CC-161.

### Phylogenetic analysis of *S. gallolyticus* subspecies

**Figure 3:**
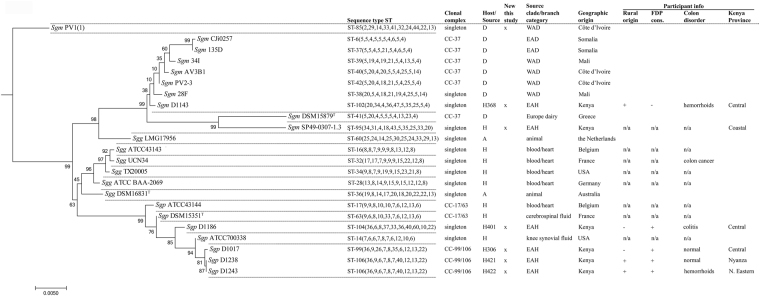
Phylogenetic MLST tree of the *S. gallolyticus* branch. Maximum likelihood phylogenetic tree of the *S. gallolyticus* branch extracted from the overall rooted SBSEC MLST tree of the concatenated sequences of 10 MLST loci. New isolate additions were: *Sgm* PV1(1), *Sgm* SP49-0307-1.3, *Sgm* D1143, *Sgp* D1017, *Sgp* D1186, *Sgp* D1238 and *Sgp* D1243. The horizontal bar at the bottom indicates the evolutionary distance in the same units as used for branch length. Isolate information is given for host/source of animal (A), dairy (D), or human (H) origin followed by participant ID for isolates obtained from colonoscopy patients of this study. “n/a” indicates that this information is not available for this strain. Participant information was only filed for human samples. Sequence type (ST) and clonal complex (CC) assignment was indicated based on MLST profile analysis.

A total of seven new *S. gallolyticus* isolates and STs were added to the SBSEC MLST scheme (Figs 1 and 3). The three *Sgm* isolates comprised of one WAD and two EAH isolates clustered clearly distinct. They did not match existing ST or CC definitions and therefore formed singletons. *Sgm* PV1(1) (WAD) branched as a single isolate and separated from the main *S. gallolyticus* clade. Within the main *Sgm* clade, a clear division between the *Sgm* type strain clade and the majority of African *Sgm* isolates was observed using sequence-based analysis (Fig. 3). However, profile-based analysis assigned the majority of EAD and WAD *Sgm* to CC-37, together with the *Sgm* type strain. *Sgm* CC-37 was defined around ST37, although no direct founder was predicted. CC-37 was comprised of six isolates each representing a unique ST, but all members of CC-37 shared a dairy origin suggesting CC-37 as a main dairy CC for *Sgm*.

EAH *Sgp* isolates formed subclades with *Sgp* ATCC700338 and *Sgp* DSM15351^T^. CC definition among *Sgp* was only possible for two pairs while the other STs remained singletons. *Sgg* represented only singletons without any CCs. This finding of a majority of singleton isolates was also reflected in highest SID values of 1 for *Sgm*, *Sgg* and *Sgp* indicating absolute diversity (Supplementary Table S1). DNA sequence identity across the 10 alleles showed high conservation of 99.2 and 99.5% for *Sgg* and *Sgp*, respectively, whereas *Sgm* featured 98.4% (Supplementary Table S2).

### Distribution of *Sii, Sgm* and *Sgp* isolates in relation to colonic disorders and consumption of traditionally fermented dairy products

A total of 40 SBSEC isolates originating from 33 colonoscopy patients were analysed. Of these, EAH *Sii* isolates (n = 34) from 30 different colonoscopy patients were integrated into the MLST scheme (Supplementary Fig. S1). These 34 *Sii* isolates were obtained from eight participants with colorectal cancer (CRC) (8/34 isolates), 12 participants with haemorrhoids (14/34 isolates) and nine participants with normal colons (11/34 isolates).

EAH *Sii* were distributed among clade (i), with the exception of *Sii* D1266 clustering in clade (ii) yielding a clear separation for most EAH *Sii* isolates from those of blood origin of clade (ii)^24^ and EAD *Sii* of clade (vii) (Supplementary Fig. S1). There was no apparent allocation of diagnoses as well as normal colon types or patient origin to specific subclades within clade (i) due to a high degree of sequence identity (Supplementary Fig. S1). Isolate D1266 of a patient suffering from haemorrhoids clustered among these presumptive pathogens of clade (ii).

Consumption of FDP was a rare trait among participants and only recorded for seven patients (Fig. 3 and Supplementary Fig. S1). This included two exclusive *Sii* carriers, three exclusive *Sgp* carriers and two carriers of either *Sii* and *Sgp* or *Sii* and *S. lutetiensis*. None of these isolates of FDP consumers were found to harbour dairy adaptation markers *lacS* or *lacZ*. Furthermore, MLST clustering of *Sii* and *Sgm* of these EAH isolates was clearly distinct from the corresponding species among the dairy adapted EAD isolates, indicating that EAH *Sii* and possibly also *Sgm* represent different lineages from EAD *Sii* and *Sgm*, respectively.

For *Sgm*, *Sgp* and *S. lutetiensis*, none of the patients reported to carry *Sgm* (one patient, one isolate), *Sgp* (four patients, four isolates) or *S. lutetiensis* (one patient, one isolate) were diagnosed with CRC, whereas haemorrhoids was the most common finding in three patients, followed by normal colon in two patients (Fig. 3).

## Discussion

Sub-Saharan Africa has unique lineages of dairy adapted *Sii* isolated from traditional FDP that are clearly distinct from human and animal isolates obtained from Europe or Asia^24^. Previous epidemiologic data of patients undergoing colonoscopy at KNH suggested an association between CRC and *Sii* as well as haemorrhoids and *Sii*. Although no causality is proven, this finding raises concerns considering the large population consuming *Sii* on a daily basis via FDP. However, before this study, no African human *Sii* isolates had been analysed to reveal the population structure, phylogenetic relationships and potential evolution of African human *Sii* vs other human and particularly dairy *Sii*. In addition, this study reports the first human *Sgm* and *Sgp* isolates obtained in Africa to give a more comprehensive insight into SBSEC in Africa.

A total of 83 new SBSEC isolates including 75 *Sii* isolates of human, dairy and animal origins were therefore included in this study for a total of 157 SBSEC isolates in the MLST scheme. Of these 75 *Sii*, 34 *Sii* isolates originated from human patients undergoing colonoscopy at KNH, Kenya while others were obtained from Kenyan infants as well as food and human sources in Africa, Europe, Asia and America. Among the *Sii*, CC-90 was clearly delineated from all other CCs, and encompassed all human blood isolates and thus potential human pathogens. CC-90 could therefore be considered as a key pathogen CC of *Sii*. The presence of single EAH and WAD *Sii* among CC-90 raises concerns for human carriers and food products containing these strains. However, the clear delineation of CC-90 from the EAD CC-71 and EAH clade (vii) as well as most other WAD/EAH CCs and clades supports further in-depth functional and genomic comparisons between key representative isolates of these CCs.

The unexpectedly close relationship between EAH *Sii* and WAD *Sii* raised further concerns and questions. The EAH *Sii* isolates were, with the exception of one isolate, closest related to WAD *Sii* isolates and clearly distinct from EAD *Sii* isolates. The majority of EAH and WAD *Sii* isolates clustered in shared CC-101 and CC-161 instead of CC-71 of EAD. This phylogenetic division was related to highly similar or shared alleles in five out of ten loci, suggesting a common ancestor for most EAH and WAD *Sii*. Furthermore, EAH and WAD *Sii* isolates shared the unique feature of *thrS* alleles with highest sequence identity to *S. thermophilus/S. salivarius*. This could be a result of genetic exchange at the base of this clade to define this new lineage. As observed for *Sgp* and *Sgm* in comparison to *Sgg*, this might further suggest a common ancestral *Sii*, possibly of human or animal origin, from which these lineages diverged and undergo niche-adaptation, particularly for EAD *Sii*^7,34^.

The combined clustering of EAH *Sii* and WAD *Sii* isolates also raised questions regarding the original reservoir of these lineages as well as the possibility for regular exchange of isolates between humans and dairy products. EAH isolates were obtained from several participants consuming traditional FDP. Kenyan FDP were observed to contain dairy adapted *Sii* in over 90% of samples, of which strains clustered exclusively in the EAD *Sii* clade (vii)^21,24^. The absence of the dairy adaptation marker genes *lacS/lacZ* in all EAH *Sii* isolates as well as a clear MLST separation from EAD *Sii* isolates strongly suggests that none of the EAH *Sii* isolates originated from FDP. This also suggests that the observed association between EAH *Sii*^27^, haemorrhoids and CRC does not directly apply to the EAD *Sii* lineage without further strain-to-strain evaluation.

Whether the reason for the absence of EAD *Sii* in FDP consumers is related to the ability of EAD *Sii* to compete against intestinal *Sii* and then colonize the gastrointestinal tract remains to be investigated. Colonization is often related to an initial adhesion step. Adhesion to extracellular matrix proteins of the gut such as mucin was limited among EAD in contrast to human isolates^24^, and could be an indication of reduced competitiveness within the gut microbiota. This will of course require further functional comparisons between isolates and additional field-based studies to directly link dairy product analysis with consumer stool analysis, as well as comparisons between East and West Africa.

This East-West comparison seems crucial due to the different dairy *Sii* lineages and potentially different human *Sii* lineages. It also has important safety implications, as the clear division between the EAH/WAD *Sii* lineages of clade (i), the potential pathobiont human *Sii* lineage of clade (ii) and the dairy adapted EAD lineage of clade (vii) might enable better definition of possible virulence factors and dairy marker genes for a thorough safety assessment. However, this also means that food isolates (WAD, Asian and Mexican *Sii*) clustered in clades (i) and (ii) will require even more detailed examinations to provide recommendations for fermented food production.

The causality of SBSEC to CRC is an ongoing investigation^9^. In this study, no apparent link between EAH *Sii* isolates, geographic patient origin and CRC or haemorrhoids could be established (Supplementary Fig. S1). This was certainly limited by the available number of strains for analysis. It is however an important step towards establishing better epidemiologic data for *Sii*. *Sii* showed an association with CRC and haemorrhoids among colonoscopy patients at KNH, Nairobi, Kenya^27^. This finding therefore supports the recommendation that the detection of any SBSEC member justifies colorectal examination^8^. However, these EAH *Sii* were not related to EAD *Sii*, and thus disease associations found for EAH in colonoscopy patients might not apply to EAD *Sii*. Further epidemiologic and comparative investigations are required to determine the role of EAD *Sii* lineage strains in relation to EAH and WAD, particularly among larger sample numbers of FDP consumers. These assessments will also need to consider recent findings relating to rotavirus vaccine responses among Ghanaian infants, which showed positive correlation with SBSEC titres in gut microbiota^35^, and could thus further highlight a special role of SBSEC members in sub-Saharan Africa.

Within the *S. gallolyticus* branch, *Sgm* lineages provided several indications for unexpected phylogenetic differentiations. *Sgm* PV1(1) isolated from WAD branched off early from all other *S. gallolyticus* members. This finding will require in-depth analysis via whole genome sequencing to determine the reasons for this branching and assist in species classification. Within the *Sgm* main clade, only one EAH isolate (SP49-0307-1.3) clustered close to the *Sgm* type strain, whereas all others formed a clearly-separated African lineage when using sequence-based analysis. However, profile-based analysis indicated the formation of a possible dairy CC-37 together with the *Sgm* type strain while EAH *Sgm* and PV1(1) represented singletons, suggesting possible diverging human and dairy lineages as for *Sii* but on a more global scale. However, the differentiation between EAH, EAD and WAD *Sgm* is less established compared to *Sii* and too early for further interpretation. In contrast, *Sgp* displayed a single African lineage clearly integrated into a separated branch and CC-99/106 within the *Sgp* clade. The number of available strains and the presence of mostly singletons for *Sgp* as well as *Sgg* are too little for further interpretation at this stage.

These different observations in MLST trees calculated from profiles (Fig. 2) vs concatenated sequences (Fig. 3 and Supplementary Fig. S1) relate to the effect of recombination as a major driver for intrastrain variations within the SBSEC^24,36^. A profile-based approach in the case of a recombinant population provides equal weight for any kind of mutation and thus reduces the impact of genetic exchange among lineages^36^. In recombinant populations, this can lead to discrepancies between CC definitions according to profiles and concatenated sequence phylogenies as observed in this study (Fig. 3 and Supplementary Fig. S1). Often, species do not evolve as strictly clonal or recombinant populations but rather feature a combination of clonal and recombinant evolution^36^. Profile and sequence-based analysis in parallel helped to reveal the *thrS*-allele influenced delineation of clade (i) among *Sii*, but also the clear definition of key CCs for EAD *Sii* (CC-71), EAH/WAD *Sii* (CC-101 and CC-161), potential human pathogens *Sii* (CC-90) as well as potential *Sgm* dairy lineage (CC-37) (Fig. 3 and Supplementary Fig. S1).

Conclusively, MLST analysis of 157 SBSEC isolates including the first set of *Sii*, *Sgm* and *Sgp* isolates obtained from patients undergoing colonoscopy at KNH, Nairobi, Kenya, revealed unexpected insights into the phylogeny and population structure of the SBSEC including multiple recombination events. Africa-specific lineages were obtained for *Sii*, *Sgm* and *Sgp* requiring in-depth comparisons to described pathogens. Among *Sii*, CC-90 was delineated as a potential main pathogen lineage comprised of all human blood isolates but also single EAH, WAD, animal and food isolates, indicating health risks for these food and faecal isolates. Unexpectedly, most EAH *Sii* isolates shared closest phylogeny, allele sequences and absence of dairy adaption markers with most WAD *Sii* isolates to form one major clade comprised of two mixed CCs and multiple singletons. This clade structure indicates a shared ancestor for EAH and WAD *Sii* isolates that is clearly distinct from the EAD *Sii*. In East Africa, this finding seems to suggest the evolution of a unique *Sii* dairy lineage, which is significantly separated from any African human *Sii* isolate, even those obtained from East African FDP consumers and colonoscopy patients. The association of EAH *Sii* with CRC and haemorrhoids should therefore not be directly linked to EAD *Sii* without further functional and genomic comparison to potentially separate pathogenic, pathobiont and food lineages. Consequently, clade- and CC-specific marker genes will need to be identified to enable a thorough safety assessment per lineage. Furthermore, detailed epidemiological data on human SBSEC isolates paired with dairy product analysis from the regular population in Africa, particularly West Africa due to the EAH-WAD *Sii* link, and other continents will be required for a more comprehensive picture beyond the hospital setting. The daily consumption of *Sii*-containing food products by approximately 200 million people in sub-Saharan Africa demands such actions given the general recent pathogenicity implication postulated for all SBSEC members^8^, but under consideration of the novel population structure findings of this study.

## Methods

### Ethical approval of the study

This study was approved in Kenya by the Kenyatta National Hospital/University of Nairobi, Ethics and Research Committee (KNH/UoN ERC)-approval number P389/07/2012. In Switzerland, the Ethics Committees of ETH Zurich and Kantonale Ethik Kommission Zurich (KEK) approved the study under decision numbers EK 2013-N-78 and KEK-StV-Nr. 47/14, respectively. Strains originating from Msambweni, Kenya, were obtained from human faecal samples covered by KNH-ERC/A/337, the University of KwaZulu-Natal (BF121/08), the ETH Zurich (EK 2009-N-53) and registered at clinicaltrials.gov (NCT01111864)^29^. Informed consent was obtained from all study participants and/or their legal guardians. The study was conducted in accordance with the Declaration of Helsinki^37^.

### Origin and overview of bacterial strains and criteria for isolate selection

This study was based on the pool of 130 SBSEC isolates obtained during the colonoscopy study performed at Kenyatta National Hospital (KNH), Nairobi, Kenya involving 273 participants recruited from 2013 until 2015^27^. The 130 isolates were obtained from 56 out of 228 unique study participants. These isolates represent the first human SBSEC isolates of African origin to be analysed for their phylogeny and evolutionary relationships. For each isolate, corresponding participant hospital diagnosis of colon disorders and responses from a guided interview on lifestyle, socio-demographics and dietary habits is available^27^.

The criteria for isolate selection among KNH human isolates for this study were based on patients’ colonic disorder, FDP consumption status, residence (urban or rural) as well as isolate rep-PCR fingerprint and presence of *lacS/lacZ* marker gene. This detailed selection was performed to achieve a wide variety of isolates with different patient backgrounds. A total of 40 SBSEC and *Sii* from 33 different patients were thus selected. Species and subspecies status of these isolates was previously determined using a 16S rRNA gene-based assay and partial *groEL* sequencing^21,24,27^. The species distribution was 34 *Sii* (30 patients), one *Sgm* (one patient), four *Sgp* (four patients) and one *S. lutetiensis* (one patient) of which one patient each was determined as combined carrier of *Sii* and *Sgm*, *Sii* and *Sgp* or *Sii* and *S. lutetiensis*.

As an outgroup to the hospital-collected samples, *Sii* (n = 7) and *Sgm* (n = 1) isolates from faecal samples of eight infants participating in a gut microbiota study in Mswambeni, Kenya were incorporated^29^. In addition, further SBSEC and *Sii* isolates were incorporated to increase the diversity for subsequent comparisons. Among these additional isolates, *Sii* and *Sgm* dairy isolates were previously collected from traditionally fermented dairy products in Kenya (*Sii* n = 18), Somalia (*Sii* n = 2) and Côte d’Ivoire (*Sii* n = 8, *Sgm* n = 1)^20,21^. Further diversity among the isolate pool was achieved through SBSEC isolates kindly donated by other research collections including single isolates from external culture collections from hospitals in Spain (n = 2; *Sii* P-9 and JIM9345)^32^, Taiwan (n = 1, *Sii* NTUH-8)^6^, animal bovine milk isolate from France (n = 1; *Sii* ANSES 6953)^33^, Italian Grana cheese (n = 1; *Sii* 42)^31^ and Mexican fermented maize (n = 1, *Sii* pozol)^30^. Overall, 83 SBSEC isolates were processed in this study and included in the SBSEC MLST scheme.

For validation of assays, the following reference strains were obtained for this study from the Culture Collection of the Laboratory of Food Biotechnology (FBT) of ETH Zurich, Zurich, Switzerland, Deutsche Sammlung von Mikroorganismen und Zellkulturen (DSMZ) (Braunschweig, Germany) and the Culture Collection of the University of Gothenburg (CCUG) (Gothenburg, Sweden): *Sii* CJ18^25^, *Streptococcus thermophilus* DSM20259 (yoghurt isolate, DSMZ), *Sgg* DSM16831^T^ ^38^, *Enterococcus faecium* DSM20477^T^^39^ and *Sii* CCUG43820^T^ ^3^.

### Growth media and growth conditions

Cultivation of SBSEC strains was performed overnight in Brain Heart Infusion broth (BHI, Biolife, Milan, Italy) at 37 ^o^C under aerobic conditions. The purity of strains was evaluated by streak plating onto Mitis Salivarius agar medium (Becton Dickinson, Allschwil, Switzerland) under aerobic incubation for 1–2 days at 37 °C. The stock cultures were stored at −80 °C in BHI broth supplemented with 33% (v/v) glycerol.

### Molecular characterization of SBSEC isolates

#### General DNA isolation procedures, PCR conditions, downstream processing and sequencing of amplified DNA fragments

DNA from single colonies was extracted by lysis and storage in an EDTA, Trizma-base, Triton-X 100-based buffer^24,40^. All PCR assays were performed using 2× concentrated PCR Master Mix (Thermo Scientific, St. Leon-Rot, Germany), 1 µM primer concentration and sterile ddH_2_O to a final volume of 20 µL^21^. All primers were obtained from Microsynth (Balgach, Switzerland). Purification of DNA amplicons was performed using the GFX PCR DNA and Gel Band Purification Kit (GE Healthcare, Buckinghamshire, UK) or direct filtration by Microsynth AG (Switzerland). Sanger sequencing of purified DNA amplicons was performed at GATC (Konstanz, Germany) and Microsynth AG (Switzerland) using the same primers as for PCR amplification.

#### Screening for dairy adaptation marker genes *lacS* and *lacZ*

All strains were subjected to *lacS* and *lacZ*-specific PCR assays to determine the presence of a *lacS/lacZ*-mediated lactose uptake system using *lacS* and *lacZ* as marker genes for dairy adaption^24^.

#### SBSEC MLST assay

SBSEC and *Sii* phylogeny was assessed using the SBSEC MLST assay^24^. A total of 83 SBSEC and *Sii* strains were processed and submitted to the public repository on pubmlst.org. Processing of DNA sequencing chromatograms was performed in CLC Genomic workbench 7.5 (Qiagen Aarhus A/S, Denmark). Sequence quality trimming was performed with a parameter setting of 0.1 followed by paired-read assembly per strain and loci. Curation, allele and ST assignment was performed as previously described using MEGA7.0 and START2^24^.

Analysis of phylogeny was performed in MEGA7.0 and START2 based on the Maximum likelihood algorithm (200 bootstrap replications) to construct phylogenetic trees using the concatenated sequence-based MLST data of all 10 alleles for all 157 SBSEC isolates.

To investigate the genetic diversity of the ten housekeeping genes and to elucidate the impact of the newly added strains, key performance indices such as d_N_/d_S_ ratio, index of association (I_A_ and I_A_^S^ for the standardized index) were calculated using START2^24^. For d_N_/d_S_, N is the number of non-synonymous sites (nucleotide substitutions change the amino acid) and S is the number of synonymous sites (nucleotide substitutions do not change the amino acid). d_N_ is the proportion of non-synonymous sites and d_S_ the proportion of synonymous sites. Investigating the d_N_/d_S_ ratio provides information about the degree of selection; in our case the degree of selection within the ten housekeeping genes. A ratio with a value less than 1 indicates that the respective gene is under stabilizing selective pressure, which means that the population mean stabilizes and the genetic diversity decreases^41^. I_A_ and I_A_^S^ quantify the amount of linkage disequilibrium between the alleles of the ten loci. The index of association has an expected value of 0, which means that no association between the different loci exists and indicates free recombination^42^. I_A_ and I_A_^S^ were calculated using a single isolate per ST to avoid bias. Clonal complex (CC) calculations were performed in Phyloviz 2.0 and eBURSTv3 for groups of STs sharing 7 or more out of 10 alleles with at least one other member of this group^24^. Simpson’s Index of Diversity (SID) was calculated for allele profiles to indicate infinite diversity for values of 1 and no diversity for values of 0^43^.

### Data availability

All data generated or analysed during this study are included in this published article and pubmlst.org. Additional information and access to raw data is available from the corresponding author on reasonable request.

## Electronic supplementary material

Supplementary Info
