# Supplementary material for: Colorectal cancer-associated Streptococcus infantarius subsp. infantarius differ from a major dairy lineage providing evidence for pathogenic, pathobiont and food-grade lineages
Source: Sci Rep. 2018 Jun 15;8:9181. doi: 10.1038/s41598-018-27383-4 (PMC6003927; doi:10.1038/s41598-018-27383-4)
Supplement: Supplementary file 1 — Supplementary Info [file 41598_2018_27383_MOESM1_ESM.pdf]

Colorectal cancer-associated *Streptococcus infantarius* subsp. *infantarius* differ from a major dairy lineage providing evidence for pathogenic, pathobiont and food-grade lineages

Dasel Wambua Mulwa Kaindi[1], Wambui Kogi-Makau[1], Godfrey Nsereko Lule[2], Bernd Kreikemeyer[3], Pierre Renault[4], Bassirou Bonfoh[5,6,7], Nize Otaru[8], Thomas Schmid[8], Leo Meile[8], Jan Hattendorf[6,7], Christoph Jans[8*]

Institutional affiliations

1 Department of Food Science, Nutrition and Technology, University of Nairobi, Nairobi, Kenya

2 School of Medicine, University of Nairobi, Nairobi, Kenya

3 Institute of Medical Microbiology, Virology, and Hygiene, Rostock University Medical Centre Rostock, Germany.

4 Institut National de la Recherche Agronomique, UMR 1319 MICALIS, Jouy-en-Josas, France

5 Centre Suisse de Recherches Scientifiques en Côte d'Ivoire, Adiopodoume, Côte d'Ivoire

6 Department of Epidemiology and Public Health, Swiss Tropical and Public Health Institute, Basel, Switzerland

7 University of Basel, Basel, Switzerland

8 Laboratory of Food Biotechnology, ETH Zurich, Zurich, Switzerland

*Address correspondence to Christoph Jans, christoph.jans@hest.ethz.ch

Supplementary Figure S1, Table S1 and Table S2

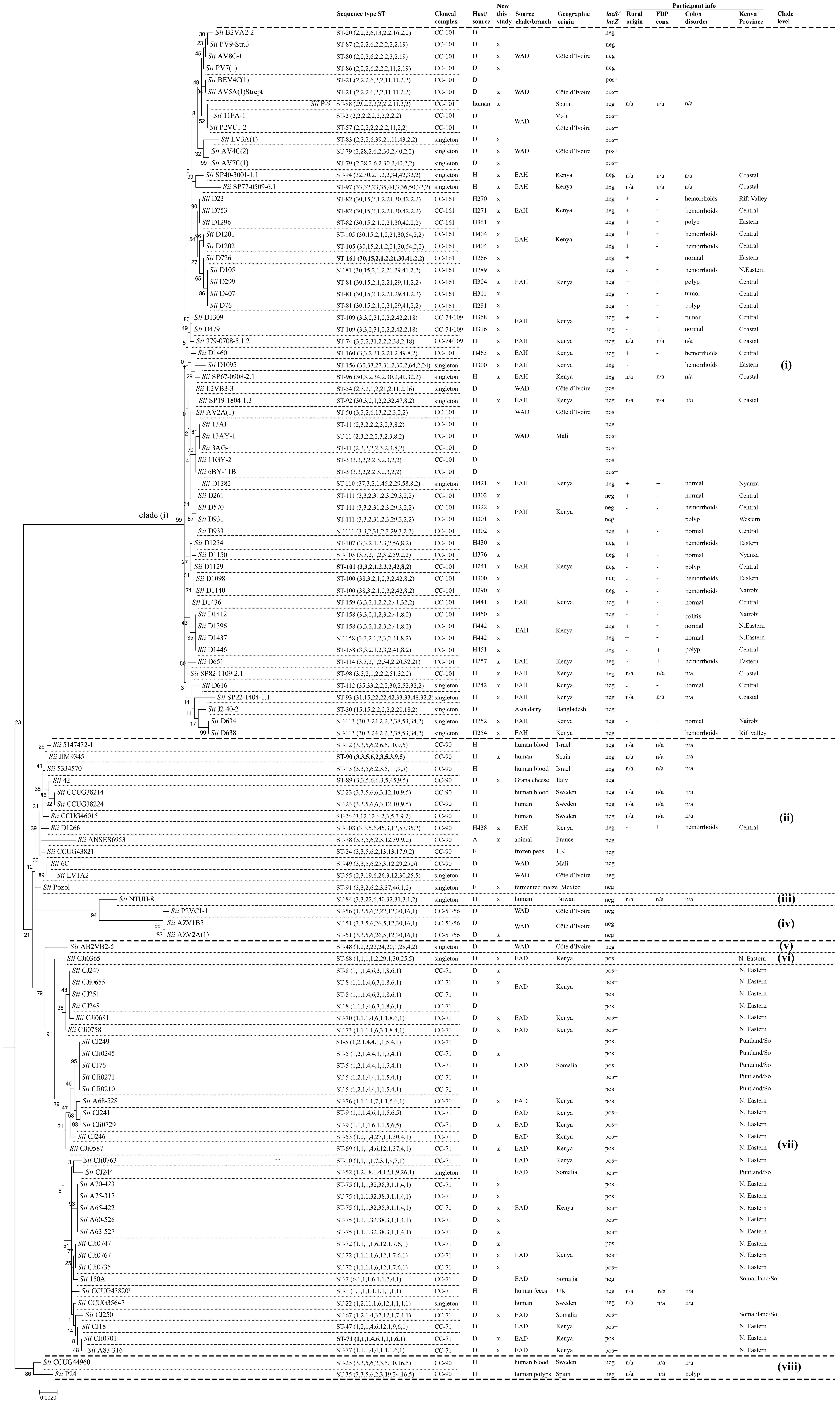

Supplementary Fig. S1: Phylogenetic MLST tree of the *S. infantarius* subsp. *infantarius* (*Sii*) branch.

Maximum likelihood phylogenetic tree of the *S. infantarius* subsp. *infantarius* (*Sii*) branch extracted from the overall rooted SBSEC MLST tree of the concatenated sequences of 10 MLST loci. Isolate information is given for host/source of animal (A), dairy (D), food (F) or human (H) origin. Geographic origin is given per clade or branch. Assignment to clonal complexes (CC) and corresponding sequence types (ST) are indicated with predicted CC-founder in bold print. The presence of *lacS/lacZ* marker genes are indicated for each isolate including isolates already included previously in the MLST scheme and those added during this study (marked as new). Participant information on rural origin, fermented dairy product (FDP) consumption, diagnosed colon disorders and geographic origin is given only for human isolates, mainly those obtained during the study conducted at Kenyatta National Hospital, Nairobi, Kenya and corresponding diagnostics as well as dietary habits. Unavailable information on external strains is indicated by “n/a”. Dashed lines indicate major clade dividers whereas dotted lines provide more detailed divisions. The length of the dotted lines corresponds to the tree hierarchy with shortest lines indicating the most detailed divisions. Longer lines indicate divisions closer to the root of the tree. The horizontal bar at the bottom indicates the evolutionary distance in the same units as used for branch length.

Supplementary Table S1: Diversity range of East African Dairy (EAD), East African Human (EAH) and West African Dairy (WAD) *Sii* isolates calculated for MLST allele profiles and sequence types (ST).

	Simpson's Index of Diversity (SID) (95% CI)[1]										
	All *Sii*	all *Sii* EAH	All *Sii* EAD	all *Sii* WAD	*Sii* clade (i) EAH+WAD	*Sii* clade (i) EAH	*Sii* clade (i) WAD	*S gallolyticus* all	*Sgm*	*Sgg*	*Sgp*
ST	0.990 (0.940-1)	0.970 (0.828-1)	0.940 (0.737-1)	0.975 (0.772-1)	0.981 (0.884-1)	0.968 (0.822-1)	0.961 (0.688-1)	0.996 (0.819-1)	1 (0.621-1)	1 (0.391-1)	0.952 (0.367-1)
ddl	0.763 (0.603-0.924)	0.662 (0.363-0.961)	0.063 (0-0.231)	0.507 (0.099-0.915)	0.745 (0.513-0.978)	0.668 (0.367-0.969)	0.294 (0-0.716)	0.901 (0.614-1)	0.644 (0.02-1)	1 (0.391-1)	0.667 (0-1)
gki	0.719 (0.550-0.889)	0.535 (0.223-0.847)	0.417 (0.070-0.764)	0.583 (0.178-0.989)	0.629 (0.374-0.884)	0.544 (0.228-0.859)	0.627 (0.164-1)	0.917 (0.643-1)	0.867 (0.343-1)	0.6 (0-1)	0.571 (0-1)
glnA	0.659 (0.480-0.837)	0.273 (0-0.549)	0.063 (0-0.231)	0.359 (0-0.746)	0.166 (0-0.36)	0.236 (0-0.502)	0 (0-0)	0.798 (0.444-1)	0.2 (0-0.686)	0.6 (0-1)	0.714 (0-1)
mutS	0.792 (0.638-0.946)	0.587 (0.278-0.896)	0.599 (0.250-0.948)	0.543 (0.136-0.951)	0.727 (0.49-0.964)	0.565 (0.251-0.88)	0.569 (0.099-1)	0.925 (0.659-1)	0.867 (0.343-1)	0.6 (0-1)	0.667 (0-1)
mutS2	0.645 (0.465-0.825)	0.188 (0-0.429)	0.724 (0.400-1)	0.551 (0.143-0.958)	0.197 (0-0.405)	0.146 (0-0.367)	0.307 (0-0.735)	0.933 (0.674-1)	0.8 (0.232-1)	0.933 (0.254-1)	0.667 (0-1)
pheS	0.795 (0.642-0.948)	0.746 (0.468-1)	0.690 (0.357-1)	0.783 (0.429-1)	0.74 (0.506-0.974)	0.694 (0.396-0.991)	0.686 (0.236-1)	0.826 (0.486-1)	0.378 (0-0.977)	0.6 (0-1)	0.524 (0-1)
proS	0.793 (0.640-0.947)	0.728 (0.445-1)	0 (0-0)	0.572 (0.166-0.979)	0.627 (0.372-0.883)	0.714 (0.423-1)	0.294 (0-0.716)	0.862 (0.544-1)	0.511 (0-1)	0.8 (0.03-1)	0.571 (0-1)
pyrE	0.952 (0.865-1)	0.890 (0.679-1)	0.843 (0.569-1)	0.859 (0.547-1)	0.909 (0.746-1)	0.885 (0.667-1)	0.784 (0.371-1)	0.858 (0.538-1)	0.733 (0.134-1)	0.933 (0.254-1)	0.286 (0-0.936)
thrS	0.846 (0.708-0.984)	0.689 (0.396-0.982)	0.605 (0.257-0.953)	0.594 (0.19-0.998)	0.588 (0.329-0.847)	0.673 (0.373-0.973)	0.294 (0-0.716)	0.858 (0.538-1)	0.533 (0-1)	0.8 (0.03-1)	0.476 (0-1)
tpi	0.643 (0.463-0.823)	0.228 (0-0.488)	0.175 (0-0.441)	0.594 (0.19-0.998)	0.285 (0.049-0.521)	0.233 (0-0.498)	0.386 (0-0.839)	0.866 (0.551-1)	0.644 (0.02-1)	0.533 (0-1)	0.571 (0-1)

[1)] CI=confidence interval

Supplementary Table S2: Identity range of East African Dairy (EAD), East African Human (EAH) and West African Dairy (WAD) *Sii* isolates calculated for concatenated sequences of all 10 alleles per isolate.

	DNA sequence identity of all 10 MLST loci[1]										
	All *Sii*	all EAH	all EAD	all WAD	clade (i) EAH+WAD	clade (i) EAH	clade (i) WAD	*S gallolyticus* all	*Sgm*[2]	*Sgg*[3]	*Sgp*
Mean identity %	98.6	99.6	99.8	98.7	99.7	99.7	99.8	97.9	98.4 (98.8)	99.2 (99.5)	99.5
Max identity %	100	100	100	100	100	100	100	100	99.9 (99.9)	99.8 (99.8)	99.2
Min identity %	95.6	97.6	99.5	96.6	99.4	99.4	99.6	95.6	95.6 (97.3)	98.5 (99.2)	99.2

[1)] calculated from concatenated DNA sequences of each isolate for a total sequence length of 4641bp per isolate; [2)] values in bracket for *Sgm* excluding PV1(1); [3)] values in brackets for *Sgg* excluding LMG17956.
